# Antitumor Activity of Intratumoral Ethanol Injection in an Orthotopic Pancreatic Cancer Cell Mouse Xenograft Model

**DOI:** 10.1155/2018/7149565

**Published:** 2018-02-21

**Authors:** Wen-Ying Zhang, Zhen-Dong Jin, Feng Liu, Hai-Hua Yuan, Bin Jiang

**Affiliations:** ^1^Department of Oncology, Shanghai Ninth People's Hospital, Shanghai Jiao Tong University, Shanghai, China; ^2^Department of Gastroenterology, Changhai Hospital, The Second Military Medical University, Shanghai, China

## Abstract

**Purpose:**

Pancreatic cancer is a lethal disease and usually is diagnosed at advanced stages of disease. This study assessed the effects of intratumoral ethanol injection using an endoscopic ultrasound (EUS) probe on the control of pancreatic cancer in a mouse orthotopic xenograft model.

**Materials and Methods:**

The subcutaneous and orthotopic human pancreatic cancer cell mouse xenograft models were established. Different concentrations of ethanol (0–95%) were injected into subcutaneous xenograft tumors. In the orthotopic tumor model, ethanol was injected into the tumor lesions under the guidance of a high-frequency EUS probe. Tumor volume, relative tumor volume (RTV), and histopathology were evaluated. The serum amylase level was analyzed at baseline and 24 h after treatment in the orthotopic tumor model.

**Results:**

Injection of 40–95% ethanol induced tumor necrosis in the subcutaneous tumor model, while there was no statistical difference between the RTVs of the two groups (*P* = 0.81). In the orthotopic tumor model, the RTV of the 80% ethanol treatment group was less than that of the saline injection group (*P* < 0.01); and histologically, there was a large area of necrosis observed in the 80% ethanol group. The serum amylase level was slightly elevated at 24 h after injection and returned to the baseline level at 7 days.

**Conclusion:**

Injection of 80% ethanol into xenograft tumor lesions of orthotopic pancreatic cancer resulted in tumor necrosis, and the procedure was safe and effective. Future studies will further confirm its antitumor activity as well as assess its safety and feasibility.

## 1. Introduction

Pancreatic cancer is one of the most lethal malignancies in the world and is frequently diagnosed at advanced stages of disease; thus, it has a very poor 5-year survival rate (less than 5%) [1]. Clinically, pancreatic cancer can be treated with surgery, chemotherapy, or radiation. Due to the advanced stage of disease at diagnosis, only approximately 20% of pancreatic cancer patients are subjected to radical resection of the tumor lesion by surgery followed by adjuvant chemotherapy, but even for these patients, the 5-year survival rate is still less than 20% because of tumor relapse and metastasis [2]. Most unresectable pancreatic cancers are usually treated with systemic chemotherapy, chemoradiation, and/or targeted therapy [2]. For chemotherapy, gemcitabine is considered to be the most effective drug for advanced pancreatic cancer, but the long-term disease-free survival has not translated into any advantage of overall survival for such patients [3]. Thus, it is urgently needed to diagnose pancreatic cancer early and to develop novel therapeutic modalities to control pancreatic cancer effectively.

To date, clinical endoscopic ultrasound (EUS) is used in the first step to diagnose pancreatic diseases; and more recently, EUS-guided fine needle injection (EUS-FNI) of agents also has been used to treat pancreatic cancer or to control tumor-induced pain through nerve blockade [4–7]. Agents delivered by EUS-FNI include chemotherapeutic drugs, brachytherapy, and viral vectors that directly ablate tumor lesions [4–7]. Most recently, ethanol has been used as a common ablative agent because it has several advantages, for example, inexpensive, readily available, and having the potential to ablate tissue rapidly. For example, percutaneous ethanol injection has been used to ablate renal cysts, hepatic cysts, and solid tumors (such as liver or adrenal tumors) [8–12]. Nevertheless, EUS-guided ethanol injection as a cancer therapeutic agent is superior to the percutaneous application because it offers real-time monitoring of the injection site and the injection amount in tumor lesions. EUS may also provide precise measurement of the tumor lesions and identification of the surrounding structures for readily delivering and minimizing damage to nontumor tissue and cells. Previous studies have demonstrated that EUS-guided injection of ethanol into a normal swine pancreas is safe and feasible [13–15], while EUS-guided ethanol ablation of pancreatic-cystic lesions also has been shown to be safe and effective [16–20]. To date, there are several case reports of successful ethanol ablation of pancreatic neuroendocrine tumors [21–23]. However, the role of ethanol in pancreatic cancer remains to be determined; thus, we designed the current study to investigate the antitumor effect of ethanol delivery through an EUS probe on an orthotopic human pancreatic cancer mouse model. Through evaluation of the effectiveness and safety of such a procedure, we hoped that a useful pancreatic cancer treatment strategy would be identified.

## 2. Materials and Methods

### 2.1. Cell Line and Culture

The human pancreatic cancer cell line SW1990 was obtained from The Cell Bank of Type Culture Collection of Chinese Academy of Sciences (Shanghai, China) and cultured in Dulbecco's modified Eagle's medium supplemented with 10% fetal bovine serum, 100 U/mL penicillin, and 100 *μ*g/mL streptomycin (all from Sigma, St. Louis, MO, USA) in a humidified incubator at 37°C with 5% CO_2_ and 95% air.

### 2.2. Experimental Animals

The research protocol was approved by the Ethics Committee of The Ninth People's Hospital, Shanghai Jiao Tong University School of Medicine (Shanghai, China). Athymic nu/nu male mice, aged 4–6 weeks and weighing 20–22 g, were obtained from the SLAC Laboratory Animal Co. Ltd. (Shanghai, China). The animals were housed in a specific pathogen-free environment, where cages, bedding, food, and water were autoclaved for 1 week to adapt to the new surroundings before the animal experiments.

### 2.3. Establishment of the Subcutaneous Pancreatic Cancer Cell Mouse Xenograft Model and Treatment Protocol

SW1990 cells at the exponential growth phase were harvested and resuspended in phosphate-buffered saline to a single cell suspension and then injected subcutaneously into the right flank of nude mice to establish the subcutaneous pancreatic cancer mouse xenograft model (1 × 10^6^ cells per mouse). One week after tumor cell inoculation, the mice were randomized into six groups, with eight mice per group: (a) normal saline control, (b) 20% ethanol group, (c) 40% ethanol group, (d) 60% ethanol group, (e) 80% ethanol group, and (f) 95% ethanol group. The xenograft lesions of the mice were injected once with different concentrations of ethanol or saline using a 25-gauge needle at a single site.

### 2.4. Establishment of the Orthotopic Pancreatic Cancer Cell Mouse Xenograft Model and Treatment Protocol

To establish the animal model, mice were anesthetized through intraperitoneal injection of sodium pentobarbital (50 mg/kg) and then a left lateral minilaparotomy was conducted by mobilizing the spleen to expose the pancreas. After that, 0.2 mL of SW1990 cells (1 × 10^7^ cells/mL) was injected into the parenchyma of the pancreas, and the abdominal incision was sutured using a surgical staple. Growth of the pancreatic cancer xenografts was monitored by using a high-frequency EUS probe (GF-UCT240-AL5, Olympus Co. Ltd., Tokyo, Japan). Ten days after tumor cell injection, the mice were divided randomly into two groups with 10 mice per group: (a) control group with saline injection and (b) ethanol group with 80% ethanol injection. The intratumoral injections were guided by the percutaneous high-frequency EUS probe. Specifically, after sodium pentobarbital anesthesia, supine animals were placed on a board and the abdomen was soaked carefully with sterile deionized water. The ultrasonic images were obtained using the EUS probe with a water bag and the direct contact method. Under the EUS probe guidance, agents were injected slowly into the tumor xenografts of the mice. The dose of ethanol per injection was amended according to the regression equation of liver tumor ethanol ablation: *Y* = 2.885*X*/12, where *X* is the maximal diameter of the tumor xenograft in cm and *Y* is the ethanol quantity in mL of [24] and our preexperimental data. All procedures were performed under sterilized conditions.

### 2.5. Assessment of Tumor Xenograft Volumes

Growth of subcutaneous tumor xenografts was measured using a vernier caliper, while growth of the orthotopic tumor xenografts was measured by a high-frequency EUS probe at baseline and 7 days after treatment. The tumor volume (*V*) was calculated by using the formula: *V* = *L* × *W*^2^/2, where *L* is the longest diameter of the tumor xenograft and *W* is the shortest diameter, according to a previous study [25]. The relative tumor volume (RTV) was determined using the following formula: RTV = *V*_t_/*V*_0_, where *V*_t_ is the weekly measured tumor volume and *V*_0_ is the initial tumor volume (before treatment). The antitumor activity of each treatment was determined by calculating the tumor growth index (TGI) value using the following equation: TGI (%) = *T*/*C* × 100%, where *T* is the mean RTV of the treated group and *C* is the mean RTV of the control group [26]. All mice were sacrificed 7 days after injection.

### 2.6. Assessment of Tumor Histology

After the mice were sacrificed, the tumor xenografts were excised, fixed in 10% buffered formalin, and embedded in paraffin for preparation of tissue sections (4 *μ*m). These tissue sections were then stained with hematoxylin and eosin (H&E) and evaluated under a light microscope by a pathologist.

### 2.7. Measurement of Serum Amylase Levels

Blood samples were withdrawn from the tail vein at baseline as well as 24 h and 7 days after treatment. These blood samples were then assessed for serum amylase levels using an automatic biochemical analyzer (BioAssay Systems, Hayward, CA, USA).

### 2.8. Statistical Analysis

All data were analyzed using SPSS 17.0 software (SPSS Inc., Chicago, IL, USA). The results were plotted as mean values ± standard deviation, and the data were evaluated by using one-way analysis of variance with the least-significant difference test for comparisons between groups. The data were considered statistically significant when *P* ≤ 0.05.

## 3. Results

### 3.1. Efficacy of the Ethanol Injection on the Control of Subcutaneous Pancreatic Cancer Cell Mouse Xenografts

**Figure 1:**
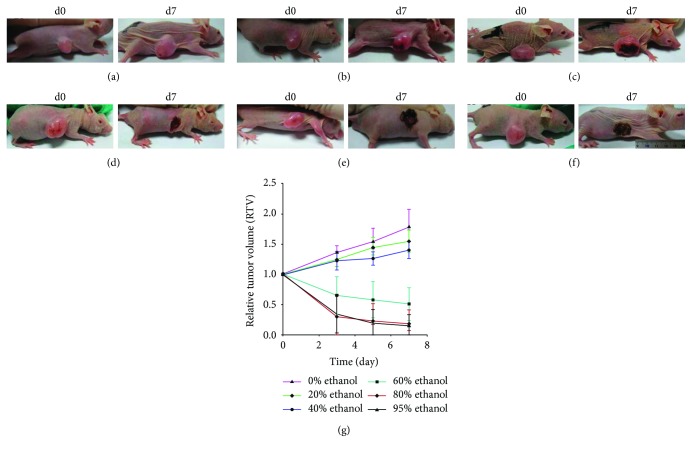
Effect of ethanol injection on the subcutaneous pancreatic cancer cell mouse xenograft model. The tumor volume and relative tumor volume (RTV) of these mouse xenografts significantly changed after different doses of ethanol were injected into the tumor xenografts of nude mice ((a) 0%, (b) 20%, (c) 40%, (d) 60%, (e) 80%, and (f) 95%). (g) Summary of the data.

**Figure 2:**
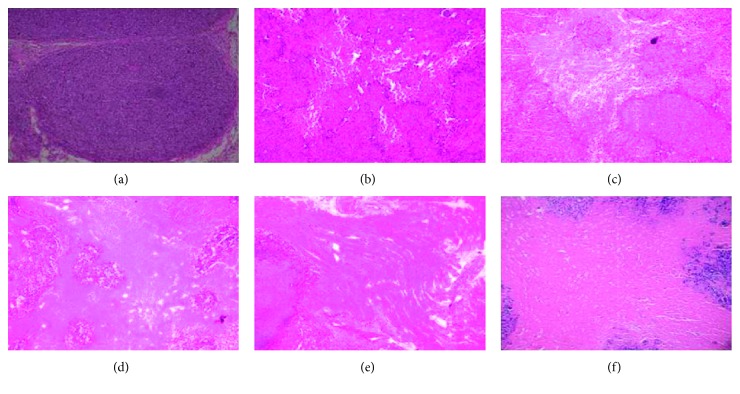
Histology of the subcutaneous pancreatic cancer cell mouse xenograft model after ethanol injection. On day 7, all mice were sacrificed and the tumor xenografts were resected for tissue processing and H&E staining. (a) 0%, (b) 20%, (c) 40%, (d) 60%, (e) 80%, and (f) 95%. The original magnification was 100x.

During the experiments, one mouse each in the 60% and 80% ethanol groups and two mice in the 95% ethanol group of the subcutaneous xenograft model died, which might have been due to the excessive ethanol dose. At 7 days after the single ethanol injection, tissue xenografts showed a large area of tumor necrosis in the mice injected with 60%, 80%, or 95% ethanol, whereas a very small area of tumor necrosis occurred in the mice injected with 20% or 40% ethanol. However, there was no necrosis present in the controls (Figure 1). The RTV of the 20% ethanol group was similar to that of the normal saline group (*P* = 0.21), whereas the RTVs of the 40%, 60%, 80%, and 95% ethanol groups were less than that of the normal saline group (*P* < 0.01); and the RTVs of the 80% and 95% ethanol groups were less than that of the 60% ethanol group (*P* = 0.003 and *P* = 0.009, resp.). However, there was no difference in the RTVs of the 80% and 95% ethanol groups (*P* = 0.819; Figure 1). The tumor growth index values were 87.1%, 78.7%, 28.7%, 10.1%, and 8.4% in the 20%, 40%, 60%, 80%, and 95% ethanol groups, respectively. H&E-stained xenograft sections also confirmed the data (Figure 2).

### 3.2. Efficacy of the Ethanol Injection on the Control of Orthotopic Pancreatic Cancer Cell Mouse Xenografts

**Figure 3:**
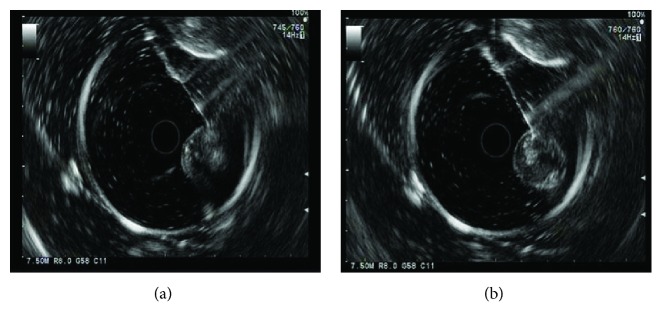
EUS-FNI of ethanol ablation of the orthotopic human pancreatic cancer cell mouse xenografts. (a) Preinjection. To ablate the orthotopic pancreatic cancer cell mouse xenograft, a fine needle was inserted into the xenograft lesion. (b) Postinjection. 80% ethanol was injected into the xenograft. Immediately after the injection of 80% ethanol, a hyperechoic area was noted in the tumor lesion.

**Figure 4:**
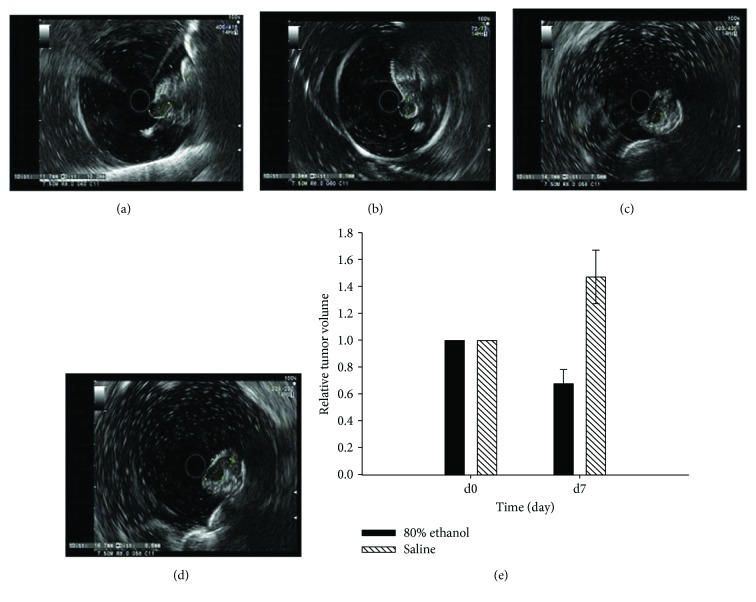
Effects of ethanol injection on the control of orthotopic pancreatic cancer cell mouse xenografts. (a) Tumor volume on day 0 after ethanol injection. (b) Tumor volume on day 7 after ethanol injection. (c) Tumor volume on day 0 after saline injection. (d) Tumor volume on day 7 after saline injection. (e) Summary of the data.

During the experiments, two mice died at 24 h and 72 h after the ethanol injection. The lethal rate was 20% in the experimental group. Immediately after the injection of 80% ethanol, a hyperechoic area was noted in the tumor xenografts (Figure 3).  Figure 4 shows representative ultrasonic images of 0 and 7 days after the ethanol or saline injection. The data showed that the tumor volume was significantly reduced from 0.82 ± 0.24 cm^3^ on day 0 to 0.60 ± 0.22 cm^3^ on day 7 in the ethanol-injected mice, whereas the tumor volume was significantly increased from 0.55 ± 0.13 cm^3^ on day 0 to 0.81 ± 0.18 cm^3^ on day 7 in the saline control mice. The RTV of the 80% ethanol group was less than that of the saline control group (*P* < 0.01). In addition, the tumor growth index was 49.7% in the ethanol group.

**Figure 5:**
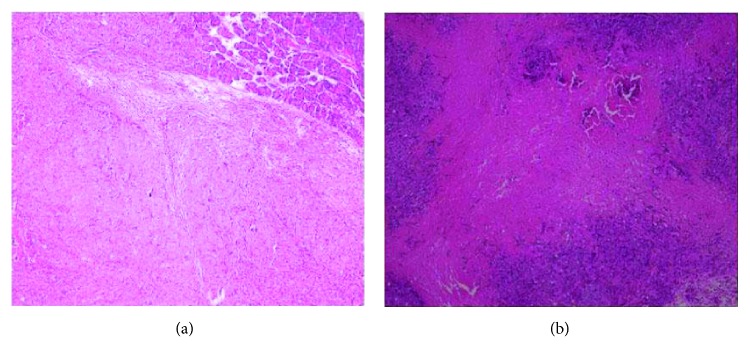
Histology of the orthotopic pancreatic cancer cell mouse xenograft model after ethanol injection. On day 7, all mice were sacrificed and the tumor xenografts were resected for tissue processing and H&E staining. (a) Saline injection. (b) 80% ethanol injection. Severe coagulation and necrosis occurred in the tumor xenografts after the 80% ethanol injection.

Furthermore, representative H&E-stained tumor xenograft sections showed that on day 7 after the ethanol injection, the tumor xenografts had a large area of necrosis in the 80% ethanol group, but there was no necrotic or damaged region in the control group (Figure 5). In addition, stomach damage did not occur in all mice, but the pancreas, liver, and spleen had varying levels of damage. For example, the pancreatic gland showed infiltration of inflammatory cells, while the liver and spleen had infiltration of inflammatory cells and large areas of necrosis.

**Figure 6:**
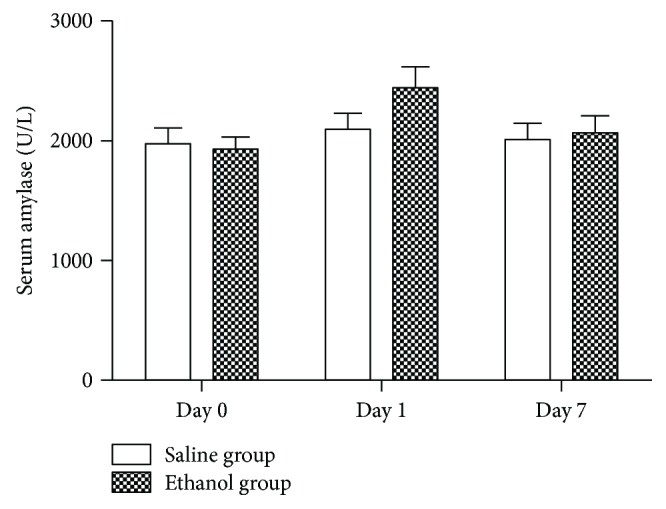
Change in serum amylase levels after ethanol injection into the orthotopic pancreatic cancer cell mouse xenografts. Blood samples were collected from the mouse-tail vein on days 0, 1, and 7 after ethanol injection and then assessed for serum amylase levels.

The serum amylase level was just slightly elevated in mice at 24 h after the 80% ethanol injection and returned to the baseline level on day 7 after treatment (Figure 6). However, there is no statistical difference compared with that of the control group (*P* > 0.05).

## 4. Discussion

To date, treatment of advanced pancreatic cancer with chemoradiation therapy is usually ineffective, and novel strategies are urgently needed to control this deadly disease and to improve the survival of patients. In the current study, we established an orthotopic pancreatic cancer mouse xenograft model and then assessed the effectiveness of high-frequency EUS-FNI of ethanol on ablation of pancreatic cancer cell xenografts. Our data showed that such treatment with ethanol could be a useful strategy to control pancreatic cancer. It is true that EUS has been used in clinical practice for more than 30 years since it was first developed for pancreatic disease diagnosis and staging of pancreatic malignancies. Only more recently, EUS has evolved into a useful therapeutic tool for pancreatic tumors. EUS-FNI, a successful minimally invasive approach, has been introduced as a novel technique for the local delivery of antitumor agents, including ethanol, brachytherapy, and ONYX-105 [5, 6, 16]. Ethanol, a particularly attractive agent, has been used previously to ablate hepatocellular carcinomas [11]. Recently, several experimental studies have demonstrated the feasibility and tolerance of EUS-guided ethanol injection in a normal porcine pancreas [13, 14, 27]. In addition, EUS-guided ethanol ablation has been used successfully for the treatment of pancreatic cysts and pancreatic neuroendocrine tumors [23, 28, 29]. Although ethanol has been applied to ablate normal, benign lesions of the pancreas, it has not been used for ablation of pancreatic cancer.

In the current study, we demonstrated the ethanol antitumor activity in the subcutaneous and orthotopic pancreatic cancer cell mouse xenograft models. We found that ethanol (20–95%) treatment resulted in local tumor necrosis in the subcutaneous model and that the antitumor activities of the 80% and 95% ethanol groups were much better than that of the 60% ethanol group, while there was no difference between the 80% and 95% ethanol groups. Matthes et al. [14] have reported a study of ethanol (0–100%) ablation in the normal porcine pancreas under EUS guidance. Their data showed that 40–100% ethanol resulted in a visible necrotic area and that 80% and 100% ethanol achieved the best tissue effects in the porcine pancreatic tissue. Moreover, Aslanian et al. [13] have demonstrated that 98% ethanol led to more widespread tissue damage to an unpredictable extent and a local complication of pancreatitis. In our current study, 80% ethanol was injected intratumorally into the orthotopic pancreatic cancer mouse xenograft model and achieved a similar efficacy [13, 14, 27]. Furthermore, the agents were injected intratumorally under the guidance of a percutaneous high-frequency EUS probe, and the growth of tumor xenografts was monitored by the high-frequency EUS probe. Our current study further demonstrated that such a treatment procedure is effective and relatively safe in the control of pancreatic cancer. Future studies will assess the dose and duration of treatment precisely.

However, there are possible risks of ethanol injection in the treatment of the orthotopic pancreatic cancer cell mouse xenograft model compared to that of the subcutaneous pancreatic cancer cell mouse xenograft model. Ethanol may induce pancreatitis to a certain degree and injure the surrounding tissues, which are more difficult to manage than in the subcutaneous model. Our current data showed that the serum amylase level was just slightly elevated in mice at 24 h after 80% ethanol injection and returned to the baseline level on day 7 after treatment. In addition, 80% ethanol did induce local complications, such as necrosis in the liver and spleen. In other studies, Gan et al. [16] have reported the EUS-guided ethanol lavage and ablation of pancreatic cystic lesions and their data showed that the procedure was able to reduce tumor lesions effectively, without complications such as pancreatitis in the short- and long-term follow-up periods. Moreover, Levy et al. [30] have described five patients with insulinoma who received EUS-guided ethanol ablation, and there were no complications observed during or after the EUS-guided procedure. Therefore, the careful and precise delivery of such a procedure is the key to eliminate unwanted side effects, like damage of other tissues and cells. In our current study, the complications might have been induced by extravasation of the injected ethanol. Thus, further improvements of injection devices are required. Furthermore, although the EUS-FNI technique has been used for the local delivery of antitumor agents, a single injection is definitely insufficient. Ohara et al. [31] have designed multiple injectable needles, and their data showed that they increased the distribution of injected drugs compared with a single-point injection. Therefore, future studies will evaluate multiple ethanol injections to treat pancreatic cancer using EUS-FNI.

In conclusion, our current study suggests that EUS-FNI of ethanol into pancreatic cancer cell mouse xenografts is feasible, safe, and effective and that the 80% ethanol injection reduces the volume of pancreatic cancer xenografts in the orthotopic pancreatic cancer mouse model. Further studies will assess multiple injections and the time required to treat pancreatic cancer in more detailed assessments of its safety and efficacy.

## References

[1] Jemal A., Bray F., Center M. M., Ferlay J., Ward E., Forman D. (2011). Global cancer statistics. *CA: A Cancer Journal for Clinicians*.

[2] Stathis A., Moore M. J. (2010). Advanced pancreatic carcinoma: current treatment and future challenges. *Nature Reviews Clinical Oncology*.

[3] Pliarchopoulou K., Pectasides D. (2009). Pancreatic cancer: current and future treatment strategies. *Cancer Treatment Reviews*.

[4] Wiechowska-Kozłowska A., Boer K., Wójcicki M., Milkiewicz P. (2012). The efficacy and safety of endoscopic ultrasound-guided celiac plexus neurolysis for treatment of pain in patients with pancreatic cancer. *Gastroenterology Research and Practice*.

[5] Jin Z., Du Y., Li Z., Jiang Y., Chen J., Liu Y. (2008). Endoscopic ultrasonography-guided interstitial implantation of iodine 125-seeds combined with chemotherapy in the treatment of unresectable pancreatic carcinoma: a prospective pilot study. *Endoscopy*.

[6] Hecht J. R., Bedford R., Abbruzzese J. L. (2003). A phase I/II trial of intratumoral endoscopic ultrasound injection of ONYX-015 with intravenous gemcitabine in unresectable pancreatic carcinoma. *Clinical Cancer Research*.

[7] Yoon W. J., Brugge W. R. (2012). Endoscopic ultrasonography-guided tumor ablation. *Gastrointestinal Endoscopy Clinics of North America*.

[8] Bean W. J. (1981). Renal cysts: treatment with alcohol. *Radiology*.

[9] Omerovic S., Zerem E. (2008). Alcohol sclerotherapy in the treatment of symptomatic simple renal cysts. *Bosnian Journal of Basic Medical Sciences*.

[10] Larssen T. B., Jensen D. K., Viste A., Horn A. (1999). Single-session alcohol sclerotherapy in symptomatic benign hepatic cysts. *Acta Radiologica*.

[11] Livraghi T., Bolondi L., Lazzaroni S. (1992). Percutaneous etharrol injection in the treatment of hepatocellular carcinoma in cirrhosis. A study on 207 patients. *Cancer*.

[12] Xiao Y.-Y., Tian J.-L., Li J.-K., Yang L., Zhang J.-S. (2008). CT-guided percutaneous chemical ablation of adrenal neoplasms. *American Journal of Roentgenology*.

[13] Aslanian H., Salem R. R., Marginean C., Robert M., Lee J. H., Topazian M. (2005). EUS-guided ethanol injection of normal porcine pancreas: a pilot study. *Gastrointestinal Endoscopy*.

[14] Matthes K., Mino-Kenudson M., Sahani D. V., Holalkere N., Brugge W. R. (2007). Concentration-dependent ablation of pancreatic tissue by EUS-guided ethanol injection. *Gastrointestinal Endoscopy*.

[15] Matthes K., Mino-Kenudson M., Sahani D. V. (2007). EUS-guided injection of paclitaxel (OncoGel) provides therapeutic drug concentrations in the porcine pancreas (with video). *Gastrointestinal Endoscopy*.

[16] Gan S. I., Thompson C. C., Lauwers G. Y., Bounds B. C., Brugge W. R. (2005). Ethanol lavage of pancreatic cystic lesions: initial pilot study. *Gastrointestinal Endoscopy*.

[17] Oh H.-C., Seo D. W., Lee T. Y. (2008). New treatment for cystic tumors of the pancreas: EUS-guided ethanol lavage with paclitaxel injection. *Gastrointestinal Endoscopy*.

[18] Oh H.-C., Seo D. W., Song T. J. (2011). Endoscopic ultrasonography-guided ethanol lavage with paclitaxel injection treats patients with pancreatic cysts. *Gastroenterology*.

[19] DeWitt J., McGreevy K., Schmidt C. M., Brugge W. R. (2009). EUS-guided ethanol versus saline solution lavage for pancreatic cysts: a randomized, double-blind study. *Gastrointestinal Endoscopy*.

[20] DiMaio C. J., DeWitt J. M., Brugge W. R. (2011). Ablation of pancreatic cystic lesions: the use of multiple endoscopic ultrasound-guided ethanol lavage sessions. *Pancreas*.

[21] Deprez P. H., Claessens A., Borbath I., Gigot J. F., Maiter D. (2008). Successful endoscopic ultrasound-guided ethanol ablation of a sporadic insulinoma. *Acta Gastroenterologica Belgica*.

[22] Muscatiello N., Salcuni A., Macarini L. (2008). Treatment of a pancreatic endocrine tumor by ethanol injection guided by endoscopic ultrasound. *Endoscopy*.

[23] Vleggaar F., bij de Vaate E., Valk G., Leguit R., Siersema P. (2011). Endoscopic ultrasound-guided ethanol ablation of a symptomatic sporadic insulinoma. *Endoscopy*.

[24] Lin L.-W., Lin X.-Y., He Y.-M. (2004). Experimental and clinical assessment of percutaneous hepatic quantified ethanol injection in treatment of hepatic carcinoma. *World Journal of Gastroenterology*.

[25] Ware J. L., DeLong E. R. (1985). Influence of tumour size on human prostate tumour metastasis in athymic nude mice. *British Journal of Cancer*.

[26] Medioni J., Leuraud P., Delattre J. Y., Poupon M.-F., Golmard J.-L. (2012). New criteria for analyzing the statistical relationships between biological parameters and therapeutic responses of xenografted tumor models. *Contemporary Clinical Trials*.

[27] Matsumoto K., Yamao K., Okubo K. (2008). Endoscopic ultrasound-guided ethanol injection in the pancreas in a porcine model: a preliminary study. *Journal of Gastroenterology and Hepatology*.

[28] Jürgensen C., Schuppan D., Neser F., Ernstberger J., Junghans U., Stölzel U. (2006). EUS-guided alcohol ablation of an insulinoma. *Gastrointestinal Endoscopy*.

[29] Muscatiello N., Nacchiero M., Della Valle N. (2008). Treatment of a pancreatic endocrine tumor by ethanol injection (PEI) guided by endoscopic ultrasound. *Endoscopy*.

[30] Levy M. J., Thompson G. B., Topazian M. D., Callstrom M. R., Grant C. S., Vella A. (2012). US-guided ethanol ablation of insulinomas: a new treatment option. *Gastrointestinal Endoscopy*.

[31] Ohara K., Kohno M., Horibe T. (2013). Local drug delivery to a human pancreatic tumor via a newly designed multiple injectable needle. *Molecular and Clinical Oncology*.

